# Vaccination contre le paludisme

**DOI:** 10.48327/mtsi.v3i2.2023.325

**Published:** 2023-05-03

**Authors:** Marie MURA

**Affiliations:** Unité Immunopathologie, Département Microbiologie et maladies infectieuses, Institut de recherche biomédicale des armées (IRBA), 1 place du Général Valérie André, 91220 Brétigny-sur-Orge, France; * Actes du Colloque – Centenaire de la mort d'Alphonse Laveran. 24 novembre 2022, Paris / Proceedings of the Conference – Centenary of the death of Alphonse Laveran. 24 November 2022, Paris

**Keywords:** Alphonse Laveran, Paludisme, Vaccination, Prévention, RTS,S/AS01, *Plasmodium falciparum*, OMS, Afrique subsaharienne, Alphonse Laveran, Malaria, Vaccination, Prevention, RTS,S/AS01, *Plasmodium falciparum*, WHO, Sub-Saharan Africa

## Abstract

L'espoir d'une vaccination contre le paludisme commence à se dessiner concrètement depuis 2015 avec l'avis favorable de l'Agence européenne du médicament sur un premier vaccin antipaludique, RTS,S/AS01. Six ans plus tard, l'Organisation mondiale de la santé (OMS) conseille un large déploiement de ce vaccin en Afrique subsaharienne et dans les régions à transmission forte et modérée où circule *Plasmodium falciparum.* Cette décision fait suite aux résultats favorables du programme pilote réalisé au Ghana, au Kenya et au Malawi sur plus de 800 000 enfants depuis 2019. Cet article décrit les objectifs et les principaux candidats vaccinaux ciblant les différents stades de développement du parasite, en soulignant les progrès et les limites de ces différentes approches. L’épopée RTS,S a été une étape clé dans le développement vaccinal, avec un vaccin de première génération recommandé par l'OMS chez les enfants de plus de 5 mois en Afrique subsaharienne et dans d'autres régions où la transmission du paludisme à *P. falciparum* est modérée ou forte, en association avec les autres mesures de prévention. Les efforts de recherche continuent pour mieux comprendre les corrélats de protection. Grâce aux progrès des plateformes vaccinales, de nouvelles approches multi-antigéniques, multistades, voire multi-espèces pourraient voir le jour et éclaircir l'horizon de la lutte contre le paludisme.

## Abstract (en)

Vaccination against malaria is an old dream that reemerged in 2015 with the European Medicines Agency's favourable opinion on a first antimalarial vaccine, RTS,S/ AS01. Six years later, the World Health Organization (WHO) is advising a wide deployment of this vaccine in sub-Saharan Africa and in regions with high and moderate transmission where *Plasmodium falciparum* circulates. This follows favourable results from the pilot programme in Ghana, Kenya and Malawi involving over 800,000 children since 2019. This article addresses the objectives and main vaccine candidates targeting the different stages of parasite development, highlighting the progress and limitations of these different approaches. The RTS,S saga has been a milestone in vaccine development, with a first-generation vaccine recommended by the WHO for use in children over 5 months of age in sub-Saharan Africa and other areas of moderate to high transmission of *P. falciparum* malaria, in combination with other prevention measures. Research efforts continue to better understand the correlates of protection. With advances in vaccine platforms, new multi-antigen, multi-stage, and even multi-species approaches might emerge and brighten the horizon for malaria control.

## Introduction

La vaccination contre le paludisme est un vieux rêve d'infectiologie qui commence à se dessiner concrètement depuis 2015 avec l'avis favorable de l'Agence européenne du médicament sur un premier vaccin antipaludique, RTS,S/AS01. Six ans plus tard, l'Organisation mondiale de la santé (OMS) conseille un large déploiement de ce vaccin en Afrique subsaharienne et dans les régions à transmission forte et modérée où circule *Plasmodium falciparum.* Cette décision fait suite aux résultats favorables issus du programme pilote réalisé au Ghana, au Kenya et au Malawi sur plus de 800 000 enfants depuis 2019.

Les travaux innovateurs de Ruth Nüssenzweig à partir des années 1960 ont ouvert la voie de la vaccination antipaludique grâce à trois découvertes importantes. Tout d'abord, elle a démontré en 1967 sur modèle murin que des sporozoïtes atténués par irradiation pouvaient immuniser l'animal contre le paludisme. Cette observation a été reproduite chez l'Homme dans les années 1970 par piqûres de moustiques irradiés, mais a nécessité plusieurs décennies pour isoler et purifier les sporozoïtes atténués depuis les glandes salivaires du moustique et obtenir un produit qui puisse être conservé et injecté à l'Homme [3]. Ensuite, son équipe de la New York University School of Medicine a démontré en 1979 que les anticorps ciblant un seul antigène, la protéine circumsporozoïte (CSP), étaient protecteurs. Ces travaux sont à l'origine du développement du vaccin RTS,S [4]. Enfin, elle a révélé l'importance des lymphocytes T CD8+ dans l'immunité contre le paludisme, alors que depuis longtemps la vaccination antipaludique cherchait essentiellement à induire des anticorps.

**Figure 1:**
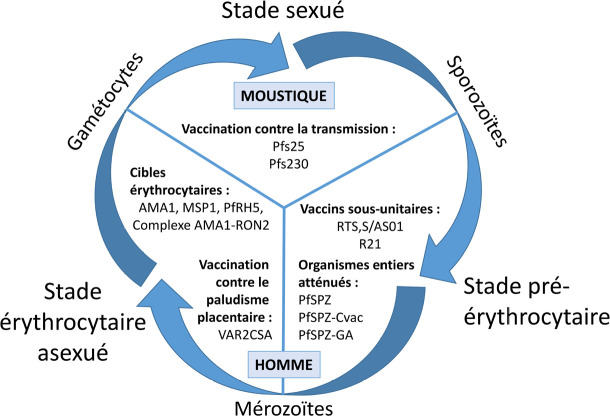
Principales stratégies vaccinales et cibles antigéniques pour le développement d'un vaccin antipaludique contre *Plasmodium falciparum* Main vaccine strategies and antigenic targets for the development of a malaria vaccine against Plasmodium falciparum

Depuis le début du xxi^e^ siècle, près de 10 essais cliniques de candidats vaccinaux antipaludiques ont lieu chaque année. Au cours des 10 dernières années, une baisse du nombre d'essais cliniques ciblant le stade érythrocytaire est observée au profit d'une augmentation des candidats vaccinaux ciblant le stade pré-érythrocytaire. Les épreuves infectieuses contrôlées chez l'Homme ont joué un rôle majeur dans la décision de « go – no go » entre les essais cliniques de phase I, dits de « sécurité », et les essais cliniques de terrain en phase II, grâce à une évaluation précoce de l'efficacité sur des volontaires sains [6]. L'objectif d'efficacité actuellement visé par l'Organisation mondiale de la santé est de 75 % à 2 ans pour *P. falciparum* et/ou *P. vivax* [5]. Nous verrons successivement les objectifs et les principaux candidats vaccinaux ciblant les différents stades de développement du parasite (Fig. 1), en soulignant les progrès et les limites de ces différentes approches.

## Vaccination PrÉ-Érythrocytaire

La vaccination pré-érythrocytaire cible les antigènes du sporozoïte de *P. falciparum,* inoculés par le moustique dans le derme, et le stade hépatique, forme cliniquement silencieuse initiée par l'invasion des hépatocytes. Cette stratégie de vaccination a pour objectif une protection contre l'infection par le parasite. En effet, en cas d’échec de cette stratégie vaccinale, les mérozoïtes libérés par le foie effectueront une amplification cyclique irréversible après invasion des globules rouges. D'un point de vue immunologique, l'objectif est d'induire des anticorps à hauts titres et haute affinité contre les antigènes de surface des sporozoïtes afin d'empêcher qu'ils puissent rejoindre le foie, ainsi que des réponses cellulaires T effectrices et mémoires résidentes tissulaires hépatiques, nécessaires à la reconnaissance puis la destruction des hépatocytes infectés. Deux grandes approches vaccinales ont été développées, sous-unitaire ou organisme entier atténué.

**Figure 2:**
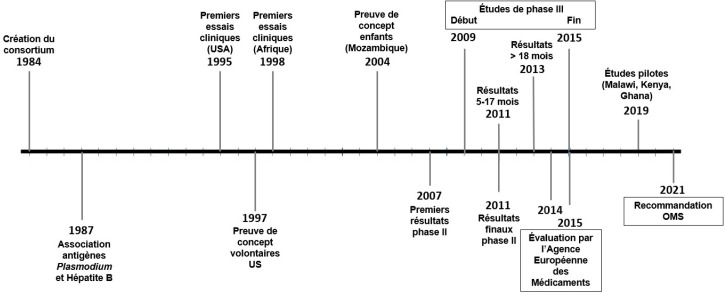
Développement du RTS,S de première génération Development of 1^st^ generation RTS,S vaccine

**Figure 3:**
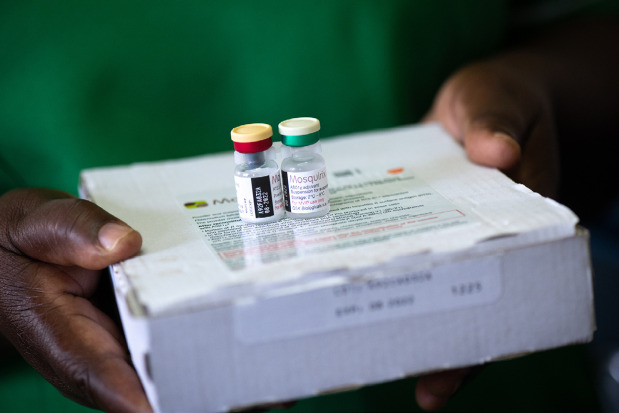
Le vaccin antipaludique RTS,S est le résultat de 35 années de recherche et de développement, en plus d’être le premier vaccin jamais mis au point contre une maladie parasitaire humaine (crédit photo : UNICEF/U.S. CDC/UN0641055/Daylin Paul) The RTS,S malaria vaccine is the result of 35 years of research and development, and is the first vaccine ever developed against a human parasitic disease. Photo credit: UNICEF/U.S. CDC/UN0641055/Daylin Paul

L'approche sous-unitaire est dominée par l'antigène CSP et le candidat vaccinal RTS,S/ AS01 (Fig. 2). Cet acronyme désigne par « R » le fragment répété de l'antigène CSP, « T » la région C-terminale comprenant des épitopes cellulaires T, fusionné à l'antigène de surface « S » de l'hépatite B, soit « RTS ». Cette protéine de fusion, exprimée en système eucaryote levure, s'associe à la protéine « S » seule pour former une particule lipoprotéique mixte « RTS,S » exprimant le fragment CSP à la surface. L'adjuvant AS01 développé par l'industriel GlaxoSmithKline finit la composition vaccinale. Les essais cliniques de phase III se sont déroulés dans 7 pays africains endémiques avec une primo-vaccination en 3 doses à un mois d'intervalle, superposée aux visites du programme élargi de vaccination, et une dose de rappel à 18 mois. La protection contre les formes cliniques, initialement de 68 % à 6 mois, était réduite à 2 ans de suivi à 36 % chez les jeunes enfants et 26 % chez les nouveau-nés, sans protection contre les formes sévères et une efficacité déclinant au cours du temps [4]. Ces résultats, très en dessous des objectifs attendus par l'OMS, ont cependant été suivis d'un programme pilote d'implémentation au Ghana, au Kenya et au Malawi pour évaluer l'intérêt de ce vaccin en association avec les autres mesures de prévention (lutte antivectorielle, chimioprophylaxie). Les principales conclusions du programme pilote sont les suivantes : (i) la vaccination est réalisable et permet d'accroître l’équité en matière d'accès aux mesures de prévention du paludisme; (ii) le profil d'innocuité du vaccin est favorable; (iii) aucun impact négatif n'a été constaté sur les autres mesures de prévention du paludisme; (iv) la réduction de l'incidence des cas graves et mortels est significative (30 %) même dans les zones où les moustiquaires imprégnées d'insecticide sont largement utilisées; (v) la vaccination est rentable dans les zones de transmission forte à modérée d'après les modélisations. Au regard de ces résultats, l'OMS recommande à ce jour l'utilisation généralisée du vaccin antipaludique RTS,S/ AS01 chez les enfants de plus de 5 mois en Afrique subsaharienne et dans d'autres régions où la transmission du paludisme à *P. falciparum* est modérée ou forte (Fig. 3).

Afin d'améliorer l'immunogénicité contre la CSP, l'Institut Jenner d'Oxford a développé un vaccin RTS,S modifié appelé R21, qui ne comprend que des antigènes de fusion « RTS » sans antigène « S » seul, en association à l'adjuvant Matrix-M développé par Novavax. Ce candidat vaccinal, testé en phase IIb sur 450 enfants de 5 à 17 mois au Burkina Faso, a atteint un taux de protection de 77 % à 1 an [2]. Cette protection a été confirmée à 2 ans de suivi [1]. L'efficacité a été observée dans une région à transmission saisonnière du paludisme avec un schéma vaccinal de 3 doses réalisé avant la saison de transmission, et un rappel à 12 mois, avant la nouvelle saison de transmission. L’étude de phase III est très attendue pour ce vaccin de deuxième génération qui, pour la première fois, atteint l'objectif de 75 % recommandé par l'OMS. L'approche « organisme entier » a montré, dès les années 1970, qu'une immunisation chez l'Homme par près de 1000 piqûres de moustiques infectés irradiés induisait une protection stérile de 50 % à 90 % dans les 12 mois suivant l'immunisation chez des volontaires naïfs. Peu exploitable en termes de manufacture vaccinale, il a fallu attendre la plateforme technique développée par Sanaria^®^ pour obtenir un produit vaccinal comportant des sporozoïtes atténués, purifiés et cryopréservés. Différentes approches permettent d'atténuer le parasite : l'irradiation (*PfSPZ Vaccine*), l'administration concomitante d'une chimioprophylaxie (*PfSPZ-CVac*) et la modification génétique par délétion de gènes nécessaires à la réalisation d'un cycle de développement complet (*PfSPZGA*). En zone d'endémie au Mali, 5 doses de 2,7.10^5^ *PfSPZ* injectés par voie intraveineuse chez des adultes induisaient une protection de 52 % à 2 ans. À l'inverse, aucune efficacité n’était constatée chez des nourrissons de 5 à 12 mois après 3 doses dans un essai clinique de phase II au Kenya. Plusieurs difficultés logistiques restent également à surmonter : la chaîne du froid nécessaire à la conservation du produit, l'inoculation intraveineuse nécessaire pour induire une protection stérile et les capacités d'intensification de la production.

## La Vaccination Éryth Rocytai Re

La vaccination érythrocytaire cible les formes non sexuées du parasite qui se multiplient cycliquement dans les globules rouges et sont à l'origine de la maladie, voire du décès dans les formes sévères. Historiquement, l'observation selon laquelle des anticorps de type IgG purifiés d'adultes africains semi-immuns éliminaient les parasites sanguins chez des enfants infectés a fait de ce stade une cible de vaccination prioritaire. Cependant, il existe plusieurs obstacles au développement d'un vaccin du stade érythrocytaire efficace. Tout d'abord, les mérozoïtes passent un temps très court (quelques secondes à peine) sous forme libre entre les globules rouges, là où ils sont accessibles aux anticorps. Ensuite, il existe un grand polymorphisme des antigènes de surface des mérozoïtes, leur permettant d’échapper à la réponse immunitaire. Les voies d'invasion des globules rouges sont redondantes. Enfin, le nombre de parasites augmente de façon exponentielle et la charge de travail pour le système immunitaire est beaucoup plus importante qu’à la phase pré-érythrocytaire. Entre les années 2000 et 2015, plus de 30 essais cliniques ciblaient le stade érythrocytaire, principalement les antigènes *AMA-1* et *MSP-1.* Ces essais cliniques n'ont pas apporté de protection contre l'infection, malgré la génération de hauts titres d'anticorps efficaces sur les tests d'inhibition *in vitro.* Actuellement, deux antigènes du stade érythrocytaire ouvrent de nouvelles perspectives vaccinales de par leur séquence conservée et leur rôle central final dans les voies d'invasion : *PhRH5* et le complexe *AMA1-RON2*.

## Autres Approches Vaccinales

Enfin, d'autres approches vaccinales sont à évoquer. **La vaccination bloquant la transmission** est une stratégie vaccinale récente qui impliquerait une adhésion collective forte, en empêchant le parasite de se reproduire au sein du moustique par la génération d'anticorps ciblant les formes sexuées du parasite. La vaccination ne protège pas l'individu vacciné mais l'entourage en empêchant la transmission du parasite par les moustiques à d'autres individus. Les chefs de file de cette stratégie vaccinale sont les antigènes *Pfs25* et *Pfs230.* **La vaccination contre le paludisme placentaire** vise à empêcher la séquestration placentaire des hématies parasitées, à l'origine des perturbations de la microcirculation placentaire. La cible vaccinale est VAR2CSA, qui lie la chondroïtine sulfate A. Enfin, des **candidats vaccinaux contre le paludisme à** ***P. vivax*** sont également en cours de développement, avec cependant un manque de moyens financiers.

## Conclusion

Ainsi, le développement d'un vaccin efficace contre le paludisme a été un long chemin semé d'embûches. L’épopée RTS,S a été une étape clé dans le développement vaccinal, avec un vaccin de première génération recommandé par l'OMS chez les enfants de plus de 5 mois en Afrique subsaharienne et dans d'autres régions où la transmission du paludisme à *P. falciparum* est modérée ou forte, en association avec les autres mesures de prévention. De plus, le vaccin RTS,S modifié, R21, est le premier candidat vaccinal à passer la barre de 75 % d'efficacité au cours d'un essai clinique en zone d'endémie. Contrairement à ce que l'on a pensé pendant des années, les candidats du stade pré-érythocytaire ont donné de meilleurs résultats de protection que les approches du stade érythrocytaire. L'approche sous-unitaire apporte aujourd'hui des résultats plus réalistes que l'approche par organisme entier atténué. Les efforts de recherche continuent pour mieux comprendre les corrélats de protection. *P. falciparum* n'est qu'une des espèces dans la lutte contre le paludisme. Or les moyens financiers manquent dans la recherche pour le développement d'un vaccin contre *P. vivax* notamment. Grâce aux progrès des plateformes vaccinales, de nouvelles approches multi-antigéniques, multi-stades, voire multi-espèces pourraient voir le jour et éclaircir l'horizon de la lutte contre le paludisme.

## Liens D'intérêts

L'autrice ne déclare aucun lien d'intérêt.
